# Supplementary material for: Assessment of Nitrate Removal Capacity of Two Selected Eukaryotic Green Microalgae
Source: Cells. 2021 Sep 20;10(9):2490. doi: 10.3390/cells10092490 (PMC8469671; doi:10.3390/cells10092490)
Supplement: Supplementary file 1 [file cells-10-02490-s001.zip › cells-1340213-supplementary.pdf]

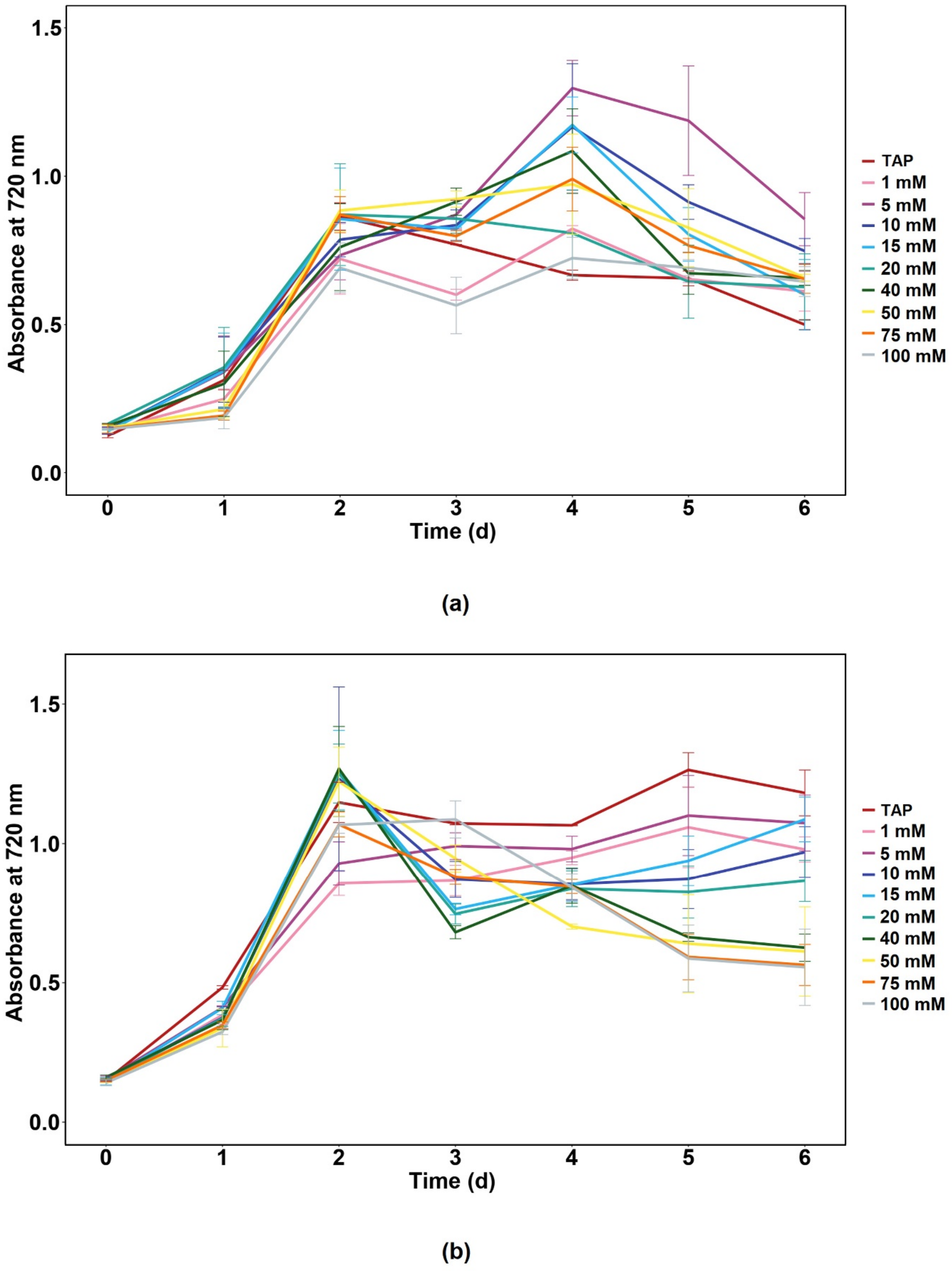

Supplementary Figure S1. Growth of *Chlamydomonas* sp. MACC-216 (**a**) and *Chlorella* sp. MACC-360 (**b**) under various concentrations of nitrate in TAP medium. Error bars are representing standard deviations.
